# Catheter ablation using pulsed‐field energy: Advantages and limitations compared with conventional energy

**DOI:** 10.1002/joa3.70011

**Published:** 2025-02-04

**Authors:** Kenji Kuroki, Hiroshi Tada

**Affiliations:** ^1^ Department of Cardiology, Faculty of Medicine University of Yamanashi Yamanashi Japan; ^2^ Department of Cardiovascular Medicine, Faculty of Medical Sciences University of Fukui Fukui Japan

**Keywords:** meta‐analysis, pulsed‐field ablation, thermal ablation

## Abstract

Atrial fibrillation (AF) poses significant risks of heart failure and stroke, emphasizing effective treatment. Catheter ablation using thermal energy sources, such as radiofrequency or cryoballoon ablation, has shown greater success in maintaining sinus rhythm compared with drug therapy. However, thermal ablation (TA) is associated with serious complications, such as atrial‐esophageal fistula, phrenic nerve palsy, and pulmonary vein stenosis. Pulsed‐field ablation (PFA) is an emerging ablation energy source that uses electroporation to selectively target cardiac tissue while sparing adjacent structures such as nerves and blood vessels. Two randomized controlled trials have demonstrated that PFA is comparable to TA in both efficacy and safety at a 1‐year follow‐up and had shorter procedure times. A review of six meta‐analyses consistently showed shorter procedural times for PFA across all studies. Additionally, three out of the four recent studies with large samples reported lower recurrence rates with PFA. Regarding complication rates, four out of four studies showed lower incidences of phrenic nerve injury with PFA, and two out of three studies reported lower rates of esophageal injury with PFA. However, four out of four studies indicated higher incidences of cardiac tamponade with PFA, highlighting the need for caution among early‐career operators. Furthermore, careful monitoring is required considering the possible unforeseen complications specific to PFA and the lack of long‐term follow‐up data. Despite these concerns, PFA shows promise as a safer, more effective, and efficient alternative to TA for AF, particularly as operator experience and device technology continue to advance.

## Graphical abstract

Pulsed‐field ablation (PFA) is safer and more efficient than radiofrequency catheter ablation. Furthermore, PFA is promising in terms of efficacy, especially considering that operators continue to gain experience and further improvements in PFA devices are expected in the future.

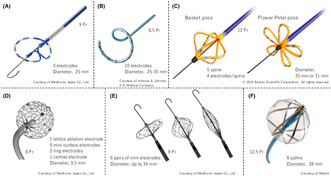

## 1 INTRODUCTION

Atrial fibrillation (AF) is a significant condition that cannot be overlooked owing to its increased risk of heart failure and cerebral infarction. Though drug therapy, including anticoagulants and antiarrhythmics, has proven effective,^1^, ^2^, ^3^ catheter ablation has been shown to be more successful in maintaining sinus rhythm.^4^, ^5^ Conventional thermal ablation (TA) using radiofrequency,^4^, ^6^ hotballoon,^7^, ^8^ cryoballoon,^6^, ^9^, ^10^, ^11^ and laser balloon^11^, ^12^ have been established as standard treatment strategies for AF, with decreasing complication rates over the past decade.^13^ However, severe complications, such as atrial‐esophageal fistula,^14^, ^15^ phrenic nerve palsy,^16^, ^17^ pulmonary vein stenosis (PVS),^18^, ^19^, ^20^ and others,^21^, ^22^ remain completely unavoidable with TA.

**FIGURE 1:**
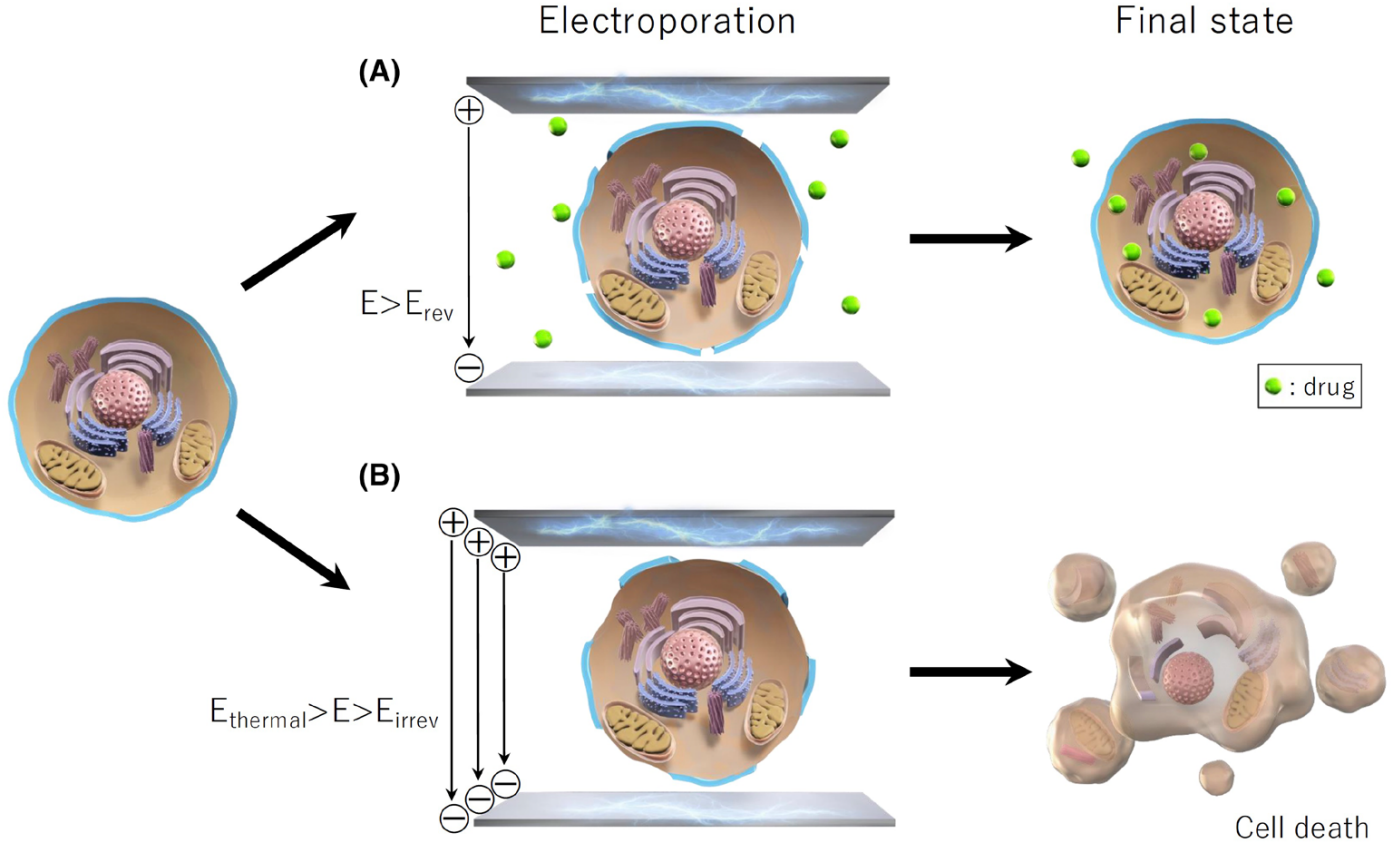
Electroporation. Depending on the intensity of application of rapid, high‐voltage pulsed electrical fields to tissue, the result can be reversible (*E*_irrev_ > *E* > *E*_rev_, where the electric field strength is controlled between the thresholds of irreversible and reversible electroporation; a technique used for gene or drug insertion into cells) (A) or irreversible (*E*_thermal_ > *E* > *E*_irrev_, where the electric field strength is controlled between the threshold of thermal effects and irreversible electroporation) (B). When the electric field is of relatively strong intensity, it may induce persistent changes in membrane permeability, leading to an irreversible breakdown of membrane structure and function and ultimately to cell death. Pulsed‐field ablation uses a multipolar electrode catheter to apply DC current with extremely short pulse widths (nanoseconds to microseconds) to target myocardial cells, causing irreversible electroporation and creating lesions. E, electric strength; irrev, irreversible; rev, reversible.

Pulsed‐field ablation (PFA) is an innovative energy source in ablation therapy, employing direct current with an ultra‐short pulse width to induce cell death through electroporation, creating pores in the cell membrane (Figure 1).^23^, ^24^ This method offers three key advantages: (1) Selectivity for cardiac tissue over other tissues such as nerves, vascular smooth muscle, endothelial cells, or red blood cells; (2) A nonthermal mechanism that preserves extracellular structures, leading to the preservation of vessels and nerves within the lesions; and (3) Tissue‐proximity dependency, eliminating the need for a strong contact‐force with the target tissue. Considering these features, PFA may be safer than TA while enhancing efficacy through sufficient energy delivery. Despite rapidly accumulating clinical evidence for PFA, most studies remain single‐arm or retrospective with limited sample sizes. Therefore, reviewing the advantages and limitations of PFA compared with the conventional TA by examining the latest randomized controlled trials (RCTs) and meta‐analyses is warranted.

## 2 ADVANTAGES OF PFA

### 2.1 Selectivity for cardiac tissue

**FIGURE 2:**
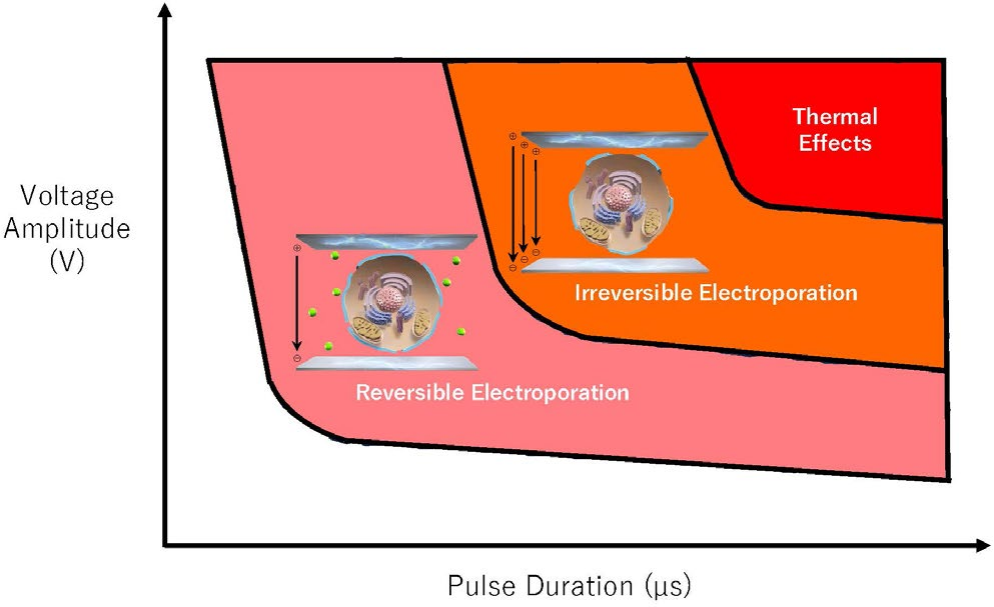
The relationship between the parameters and effects of pulsed‐field ablation (PFA). As the PFA parameters, such as the voltage amplitude and pulse duration, increase, the effects of the PFA transition from reversible electroporation to irreversible electroporation and eventually thermal effects. It is important to note that the tissue selectivity of PFA is maintained only when the parameters are appropriately adjusted to avoid thermal effects.

To fully understand the properties of the new technique, preclinical studies, including tissue assessments, are indispensable as they can thoroughly evaluate the safety and efficacy. Numerous animal studies have demonstrated the safety of PFA owing to its preferential selectivity for cardiac tissue,^25^, ^26^, ^27^ whereas TA can cause various complications by indiscriminately damaging surrounding tissues.^22^, ^23^, ^24^ Among the complications, atrial‐esophageal fistula is a rare but life‐threatening complication of TA. In an experiment ablating the esophagi of swine from the inferior vena cava (IVC), esophageal injury, including one IVC‐esophageal fistula, was observed in all cases in the radiofrequency ablation (RFA) group (*n* = 4), but not in the PFA group (*n* = 6).^25^ Animal studies also indicated that PFA has minimal impact on the phrenic nerve.^26^, ^27^ In one study, a single 200 J PFA was delivered from the right atrium of a swine to the phrenic nerve.^26^ The phrenic nerve was captured in 17/19 cases immediately after ablation and in all cases 30 min later. Follow‐up in 15 cases, 3–13 weeks later, showed no phrenic nerve issues. The tissue selectivity described above is only effective when the PFA parameters, such as the voltage amplitude and pulse duration, are appropriately adjusted (Figure 2). It is important to note that the preferential tissue selectivity for cardiac tissue can be lost if thermal effects are induced because of uncontrolled parameters.

### 2.2 Nonthermal mechanism

Pulsed‐field ablation acts on target tissue through a nonthermal mechanism when parameters are properly controlled. This mechanism results in less inflammation and greater preservation of extracellular structures compared with TA. These characteristics are likely related to the reduced incidence of PVS with PFA, as opposed to TA, whose thermal effects can lead to different complications, including PVS.^18^, ^19^, ^20^, ^28^, ^29^, ^30^ In an animal study,^31^ PFA and RFA were performed on opposite pulmonary veins in 10 swine, and the diameters of the veins were measured angiographically before ablation and 3 months later. PFA showed a 19% increase in pulmonary vein diameter, whereas RFA showed a 7% decrease. Similar results were clinically confirmed in a sub‐analysis of the IMPULSE/PEFCAT study.^32^ Furthermore, unlike thermal energy, PFA's nonthermal mechanism is not influenced by the cooling effects of the surrounding structures, which may limit the efficacy of TA. In one study, mitral isthmus ablation was performed on six swine using a lattice‐tip PFA catheter.^33^ Two weeks later, the low‐voltage linear line was maintained in all swine, and histological analysis showed a 95.5% transmurality rate in 27 sections. A clinical study using the same catheter showed that mitral isthmus block was achieved in all 14 patients within a mean time of 5.5 ± 3.5 min.^34^ Especially in the cohort where only PFA was used, PFA was delivered inside the CS in six of nine patients and with a mean ablation time of 3.5 ± 1.8 min.^34^ A potential advantage of PFA is that it can deliver energy within the CS without the risk of steam pops.

### 2.3 Tissue‐proximity dependency

The effect of PFA has been shown to depend on the distance between the electrodes and the target tissue.^35^ Therefore, it is crucial for PFA catheters to establish contact with the target tissue. However, a preclinical study demonstrated that the contact‐force does not significantly influence the lesion depth, width, or volume.^36^ Additionally, the first clinical trial (IMPULSE, PEFCAT, and PEFCAT II) reported excellent durability of the PV isolation using the optimized waveform setting without contact‐force monitoring, suggesting that PFA does not require firm contact‐force with the target tissue.^37^ Furthermore, from a safety perspective, this feature of PFA may help reduce the prevalence of cardiac tamponade in the future. Nevertheless, a relationship between the contact‐force and lesion size with PFA was observed in an animal study of ventricular ablation,^38^ and further evidence is needed to clarify this topic.

## 3 CLINICAL EVIDENCE COMPARED WITH THERMAL ABLATION

**FIGURE 3:**
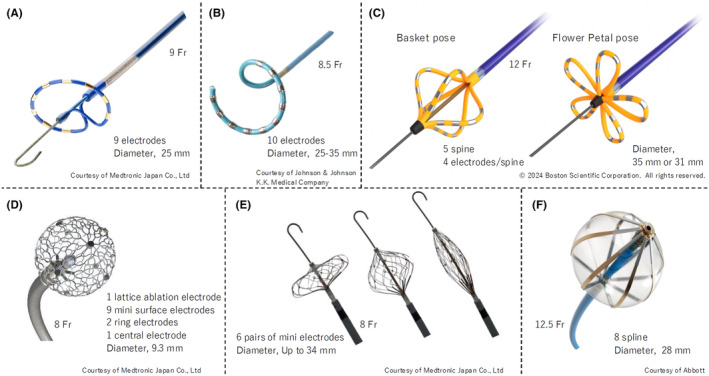
Circumferential catheters for pulsed‐field ablation. (A) PulseSelect™ (Medtronic Co., Ltd., Minneapolis, MN, USA). (B) VARIPULSE® (Biosense Webster, Inc., Irvine, CA., USA). (C) FARAWAVE™ (Boston‐Scientific, Natick, MA, USA). (D) Sphere‐9™ (Medtronic Co., Ltd.). (E) Sphere‐360™ (Medtronic Co., Ltd.). (F) Volt™ PFA Catheter, Sensor Enabled™ (Abbott, Abbott Park, IL, USA). At present, Sphere‐9, Sphere‐360, and Volt PFA catheters are not approved for sale or distribution in Japan.

Several PFA devices are now available in clinical practice or are in the investigational phase. As of October 2024, three devices—PulseSelect (Medtronic, Figure 3A),^23^ VARIPULSE (Biosense Webstar, Figure 3B),^39^ and FARAWAVE (Boston‐Scientific, Figure 3C)^37^—have received both CE mark and FDA approval. Also, in Japan, the Pharmaceuticals and Medical Devices Agency (PMDA) has approved the three devices: PulseSelect, VARIPULSE, and FARAWAVE. PulseSelect and VARIPULSE are both ring‐type catheters, but PulseSelect is positioned at the pulmonary vein ostium using an over‐the‐wire system, whereas VARIPULSE is integrated with a dedicated 3D mapping system. FARAWAVE has the longest clinical use history, having received a CE mark as early as January 2021. In fact, FARAWAVE was used as the PFA device in nearly all the studies included in the meta‐analyses discussed later. Its electrode configuration can change from a basket to a flower‐petal shape to better fit the pulmonary vein ostia. PFA catheters are large in diameter and require a large lumen sheath to perform PFA: the FARAWAVE has a larger diameter than the other two catheters (12Fr vs. 9 and 8.5Fr) and requires a larger sheath (inner lumen of 13Fr). Operators should take care to avoid drawing air bubbles into the sheath, particularly when mapping is performed using a smaller multielectrode mapping catheter.^40^ A detailed comparison of the three devices is summarized in Table 1. Sphere‐9 (Medtronic, Figure 3D)^41^ and Sphere 360 (Medtronic, Figure 3E)^42^ feature mesh‐type structures designed for point‐by‐point and one‐shot ablation, respectively. Sphere‐9 has already received CE mark approval, but Sphere 360 is still in the investigational phase. Volt (Abbott, Figure 3F) is a unique one‐shot device with eight splines on a balloon, which has just completed CE mark feasibility study and is awaiting CE mark approval.^43^

**TABLE 1:** Device specifications of the pulsed‐field ablation systems.

	Catheter shape and size	Catheter diameter (Fr)	Sheath diameter (Fr) inner/outer	Over‐the‐wire lumen	Dedicated 3D‐mapping system	Electrode size (mm)	Inter‐electrode spacing (mm)	Voltage amplitude (V)	Waveform characteristics
FARAWAVE™ (Boston‐Scientific, Natick, MA, USA)	Basket or flower‐petal, 31 or 35 mm	12	13/16.8	Yes	No	2.1	2.5	1800–2000	Biphasic/bipolar
PulseSelect™ (Medtronic Co., Ltd., Minneapolis, MN, USA)	Ring, unadjustable, 25 mm	9	10/14	Yes	No	3.0	3.8	1400–1500	Biphasic/bipolar
VARIPULSE® (Biosense Webster, Inc., Irvine, CA, USA)	Ring, adjustable, 25–35 mm	8.5	8.5/11.5	No	Yes	3.0	4.0	1800	Biphasic/bipolar

Three RCTs comparing PFA with TA have been reported (Table 2).^44^, ^45^, ^46^ In the ADVENT trial,^44^ patients with paroxysmal AF were assigned in a 2:1:1 ratio to PFA, RFA, and cryoballoon ablation (CBA), respectively (305 patients for PFA and 302 for TA). The multielectrode pentaspline ablation catheter (Farapulse; Boston‐Scientific) was used in all PFA procedures. The primary efficacy end point was defined as freedom from a composite of initial procedural failure, documented atrial tachyarrhythmia after a 3‐month blanking period, antiarrhythmic drug use, cardioversion, or repeat ablation. The primary safety end point included acute and chronic device‐and procedure‐related serious adverse events. PFA was found to be noninferior to TA in terms of both the primary efficacy and safety end points after 1 year. In another RCT comparing PFA with TA in patients with persistent AF,^45^ a dual‐energy, lattice‐tip ablation catheter was used for both PFA and RFA. The primary composite effectiveness end point, including freedom from acute procedural failure, repeat ablation at any time, arrhythmia recurrence, initiation or escalation of medication, or cardioversion after a 3‐month blanking period, was assessed after 1 year. The primary safety end point was defined as freedom from a composite of serious procedure‐related or device‐related adverse events. PFA was confirmed to be noninferior to TA in terms of both safety and effectiveness. While a shorter procedural time was demonstrated with PFA, detecting significant differences in recurrence and complication rates was difficult owing to the trial design aimed at demonstrating noninferiority. Therefore, subsequent meta‐analyses would be useful in comparing the efficacy and safety of PFA and TA. Another RCT comparing PFA with TA examined the systematic responses, including inflammation, platelet activation, and coagulation activation in the acute phase.^46^ Although peak troponin levels were significantly higher in the PFA group (10 102 ng/L [IQR: 8272–14 207 ng/L] vs. 1006 ng/L [IQR: 603–1433 ng/L]), both procedures were associated with similar levels of platelet and coagulation activation (over 50%), and the proinflammatory response 24 h after the procedure was slightly, though not significantly, higher in the RFA group. Despite causing 10 times more myocardial damage than TA, PFA had a comparable impact on platelet and coagulation activation and the inflammatory response, likely because of its distinct ablation mechanism.

**TABLE 2:** Randomized controlled trials of PFA versus TA for atrial fibrillation.

Author	Sample size	AF type	PFA device	Study design	Primary end points	Results
Reddy et al.^44^	607	Paroxysmal	Pentaspline	PFA vs. RFA/CBA	Efficacy: freedom from a composite of an initial procedural failure, documented atrial tachyarrhythmia after a 3‐month blanking period, antiarrhythmic drug use, cardioversion, or repeat ablation Safety: acute and chronic device‐ and procedure‐related serious adverse events	PFA was non‐inferior to TA with respect to both the primary efficacy and safety endpoints at 1 year
Anter et al.^45^	420	Persistent	Lattice‐tip	PFA vs. RFA	Efficacy: freedom from an acute procedural failure and repeat ablation at any time, plus arrhythmia recurrence, drug initiation or escalation, or cardioversion after a 3‐month blanking period Safety: freedom from a composite of serious procedure‐related or device‐related adverse events	Same as above
Osmancik et al.^46^	65	Paroxysmal/persistent	Pentaspline	PFA vs. RFA	Markers of myocardial damage (troponin I), inflammation (interleukin‐6), coagulation (D‐dimers, fibrin monomers, von Willebrand antigen, and factor activity), and platelet activation (P‐selectin and activated GpIIb/IIIa antigen)	Despite 10 times more myocardial damage, PFA was associated with a similar degree of platelet/coagulation activation and slightly lower inflammatory response

Abbreviations: CBA, cryoballoon ablation; PFA, pulsed‐field ablation; RFA, radiofrequency ablation; TA, thermal ablation.

Our investigation identified six meta‐analyses that compared PFA and TA in AF patients (Table 3).^47^, ^48^, ^49^, ^50^, ^51^, ^52^ In two studies, CBA alone was used as the control arm (as it is also a one‐shot device),^48^, ^50^ whereas both RFA and CBA were used in the remaining four studies.^47^, ^49^, ^51^, ^52^ All studies consistently demonstrated shorter procedural times for PFA. Furthermore, three out of the four recent studies,^49^, ^50^, ^51^, ^52^ which included large sample sizes, reported lower recurrence rates with PFA. In terms of overall complication rates, only one of six studies favored PFA.^50^ Sub‐analyses of complication rates, such as phrenic nerve injury, esophageal injury, and cardiac tamponade, were conducted in four,^48^, ^49^, ^50^, ^51^ three,^49^, ^50^, ^51^ and four^48^, ^49^, ^50^, ^51^ studies, respectively. All four studies showed comparably lower phrenic nerve injury rates between PFA and TA,^48^, ^49^, ^50^, ^51^ and two out of three studies showed lower esophageal injury rates with PFA.^49^, ^50^, ^51^ However, all four studies indicated higher rates of cardiac tamponade with PFA than with TA.^48^, ^49^, ^50^, ^51^ A recent article by Amin et al., published in the Journal of Arrhythmia (the fifth article in Table 3), analyzed 17 studies involving 2255 patients, focusing on AF and all atrial arrhythmia recurrences (AF, atrial tachycardia [AT], and atrial flutter [AFL]) separately during the follow‐up.^51^ They found that PFA significantly reduced AF recurrence but did not show a significant difference in all atrial arrhythmia recurrence, possibly indicating a higher recurrence of AT or AFL with PFA, likely because of its more proximal pulmonary vein isolation lines. Regarding complications, the study observed significantly fewer cases of phrenic nerve palsy and esophageal lesions with PFA, which was attributed to its tissue selectivity. However, an increased incidence of pericardial tamponade was noted, potentially owing to initial operator inexperience with PFA devices. Indeed, this trend has already been observed in registry trials.^53^, ^54^ The MANIFEST‐PF registry (*n* = 1568, the initial experience of the MANIFEST‐17K registry), published in 2022, reported a tamponade rate of 0.97%.^53^ In contrast, the MANIFEST‐17K registry (*n* = 17 642), published in 2023, showed a marked improvement, with a tamponade rate to 0.36%.^54^ Another meta‐analysis by de Campos et al. discussed reasons for the higher incidence of cardiac tamponade during the initial experience with PFA.^49^ They attributed the higher rate to the initial use of an exceptionally rigid guidewire for PFA catheter delivery, which resulted in inadvertent perforation of the left atrial appendage in four patients. Another contributing factor was the tight locking mechanism between the dilator and sheath, which could lead to unintended forward movement of the sheath. Despite the relatively limited experience with PFA, these meta‐analyses consistently showed benefits, including shorter procedure times, lower recurrence rates, and fewer complications.^47^, ^48^, ^49^, ^50^, ^51^, ^52^ As operators gain experience with PFA and workflows get refined, the procedure times are expected to decrease even further.^55^ However, the initial higher incidence of cardiac tamponade with PFA warrants caution. While device‐related issues have largely been resolved with updated equipment, operators must remain vigilant regarding the risk of tamponade, particularly during their initial learning curve.

**TABLE 3:** Meta‐analyses of pulsed‐field ablation versus thermal ablation for atrial fibrillation.

Author	Publication year	Journal	Included studies	Control	Sample size	Shorter procedure time	Lower recurrence rate	Lower complication rate	Lower PN injury rate	Lower esophageal injury rate	Lower cardiac tamponade rate
Aldaas et al.^47^	2023	J Interv Card Electrophysiol	6	RFA/CBA	1012	PFA	NS	NS	NR	NR	NR
Zhang et al.^48^	2024	Pacing Clin Electrophysiol	15	CBA	1880	PFA	NS	NS	PFA	NR	RFA
de Campos et al.^49^	2024	Heart Rhythm O2	18	RFA/CBA	4998	PFA	PFA	NS	PFA^a^		RFA
Rudolph et al.^50^	2024	Eur Heart J Open	11	CBA	3805	PFA	PFA	PFA	PFA	NS	RFA
Amin et al.^51^	2024	J Arrhythmia	17	RFA/CBA	2255	PFA	PFA^b^	NS	PFA	PFA	RFA
Iqbal et al.^52^	2024	Arrhythm Electrophysiol Rev	20	RFA/CBA	3857	PFA	NS	NS	NR	NR	NR

Abbreviations: CBA, cryoballoon ablation; NR, not reported; NS, not significant; PFA, pulsed‐field ablation; PN, phrenic nerve; RFA, radiofrequency ablation.

^a^ Temporary or permanent phrenic nerve palsy, esophageal injury, and atrioesophageal fistula were all included as (peri‐)esophageal injuries.

^b^ PFA demonstrated significantly reduced AF recurrence but did not show a significant difference in all atrial arrhythmia recurrence.

## 4 LIMITATIONS AND/OR UNSOLVED QUESTIONS OF PFA

This review demonstrates the safety and efficacy of PFA in the extant literature through high‐quality evidence, such as RCTs and meta‐analyses; however, there are a few limitations. First, all the PFA data in the review come from short‐term follow‐up studies. This differs from RFA literature, which has a long history and a wealth of accumulated evidence.^56^ Second, whether the optimal sequence of anticoagulation management strategy involving RFA^57^ is suitable for PFA remains unclear. Given PFA's nonthermal effects, anticoagulation protocols could be shortened. Third, while ablation strategies for persistent or recurrent AF have been well evaluated in TA,^58^, ^59^, ^60^, ^61^ PFA is limited by preliminary data.^62^, ^63^ Notably, an SVC isolation using PFA has been shown to cause no or only transient damage to the phrenic nerve in both preclinical and clinical studies.^64^, ^65^ However, potential sinus node dysfunction following a PFA delivery should continue to be carefully assessed and monitored, as PFA potentially tends to create more extensive lesions than TA. Fourth, the development of a lesion size index for PFA could enhance its efficacy and safety,^66^ similar to how lesion size indicators have been extensively studied and proven useful in RFA.^67^, ^68^ Fifth, PFA lacks data on patients with diverse clinical backgrounds. In contrast, RFA has substantial evidence across various patient populations, such as those with heart failure,^69^, ^70^, ^71^ hemodialysis,^72^, ^73^ sleep apnea syndrome,^74^ obesity,^75^, ^76^, ^77^ post‐surgical conditions,^78^ and atypical anatomical structures.^79^ Sixth, while most available data on PFA are limited to safety and efficacy, RFA has been extensively researched in terms of arrhythmia mechanisms, origins, and other aspects.^80^, ^81^, ^82^, ^83^, ^84^ Lastly, special attention should be given to PFA‐specific complications that have recently been reported in clinical practice. To date, coronary spasm and hemolysis are the two main complications specific to PFA.^85^, ^86^ The MNIFEST‐17K study, which included 17 642 patients undergoing post‐approval PFA with a pentaspline catheter, reported that the prevalence of both complications was very rare: coronary spasms occurred in 0.14% (*n* = 25) of the cases, and hemolysis‐related renal failure in 0.03% (*n* = 5). The majority of the coronary spasms (22 of 25, 88%) were proximity‐related, with clinical sequelae reported in four cases. Two cases required resuscitation and defibrillation: (1) atrioventricular block and ventricular fibrillation during PFA of the cavotricuspid isthmus, and (2) ventricular fibrillation after PFA of the right inferior PV. The remaining two cases involved chest pain in the post‐procedure recovery area, which was promptly resolved with nitroglycerin. In all patients with hemolysis‐related renal failure, the creatinine levels increased by 100% by the next post‐procedure day, and transient hemodialysis significantly improved the renal function by hospital discharge. When targeting the cavotricuspid isthmus or mitral isthmus with PFA, prophylactic administration of nitroglycerin is recommended.^85^ To minimize the risk of hemolysis, reducing the number of deliveries and ensuring proper contact with the target tissue during each delivery is advised.^86^, ^87^

## 5 CONCLUSIONS

The results of RCTs and meta‐analyses to date suggest that PFA is safer and more efficient than RFA. Furthermore, PFA is also promising in terms of efficacy, especially considering that operators continue to gain experience and further improvements in PFA devices are expected in the future. However, the safety of PFA must be carefully and continuously monitored owing to the possibility of other unforeseen PFA‐specific complications emerging in the future.

## FUNDING INFORMATION

No funding was required for this article.

## CONFLICT OF INTEREST STATEMENT

Dr. Kenji Kuroki received grants from Medtronic Co., Ltd. and Abbott Medical Japan LLC. Dr. Kuroki is a consultant for Abbott Medical Japan LLC, Microport CRM Japan Co., Ltd., and Kaneka Corporation. Dr. Hiroshi Tada received honoraria for lectures or speakers bureaus from Daiichi Sankyo Company, Ltd., Novartis Pharma K.K., Medtronic Japan Co., Ltd., Biotronik Japan, Inc., Bristol Myers Squibb, and Boston‐Scientific Japan K.K. Dr. Tada also received grants (Investigator‐initiated study unrelated to the manuscript topic) from Abbott Medical Japan LLC, Daiichi Sankyo Company, Ltd., Nippon Boehringer Ingelheim Co., Ltd., Otsuka Pharmaceutical Co., Ltd., Eli Lilly Japan K.K., and Marubun Tsusyo K.K.

## ETHICS STATEMENT

Institutional review board approval was not required as this is a review article.

## PERMISSION TO REPRODUCE MATERIAL FROM OTHER SOURCES

All material reproduced with permission.
